# Development of a multiplex qPCR assay for the simultaneous detection of *Mycoplasma bovis, Mycoplasma* species, and *Acholeplasma laidlawii* in milk

**DOI:** 10.7717/peerj.11881

**Published:** 2021-08-12

**Authors:** Kanika Chauhan, Sharif S. Aly, Terry W. Lehenbauer, Karen H. Tonooka, Kathy Glenn, Paul Rossitto, Maria L. Marco

**Affiliations:** 1 Veterinary Medicine Teaching & Research Center, School of Veterinary Medicine, University of California, Davis, Tulare, CA, United States; 2 Department of Population Health and Reproduction, School of Veterinary Medicine, University of California, Davis, Davis, CA, United States; 3 Department of Food Science and Technology, University of California, Davis, Davis, CA, United States

**Keywords:** Milk, Bovine mastitis, qPCR, Multiplex, Taqman, Diagnostic, Mycoplasma

## Abstract

Contagious bovine mastitis caused by *Mycoplasma bovis* and other *Mycoplasma* species including *Mycoplasma californicum*, *Mycoplasma bovigenitalium, Mycoplasma alkalescens, Mycoplasma arginini*, and *Mycoplasma canadense* is an economical obstacle affecting many dairy herds throughout California and elsewhere. Routine bacteriological culture-based assays for the pathogens are slow and subject to false-positive results due to the presence of the related, non-pathogenic species *Acholeplasma laidlawii*. To address the need for rapid and accurate detection methods, a new TaqMan multiplex, quantitative real-time PCR (qPCR) assay was developed that targets the 16S rRNA gene of *Mycoplasma, rpoB* gene of *M. bovis*, and the 16S to 23S rRNA intergenic transcribed spacer (ITS) region of *A. laidlawii*. qPCR amplification efficiency and range of detection were similar for individual assays in multiplex as when performed separately. The multiplex assay was able to distinguish between *M. bovis* and *A. laidlawii* as well as detect *Mycoplasma* spp. collectively, including *Mycoplasma californicum, Mycoplasma bovigenitalium, Mycoplasma canadense, Mycoplasma arginini* and *Mycoplasma alkalescens*. In milk, the lower limit of detection of *M. bovis, M. californicum*, and *A. laidlawii* with the multiplex assay was between 120 to 250 colony forming units (CFU) per mL. The assay was also able to simultaneously detect both *M. bovis* and *A. laidlawii* in milk when present in moderate (10^3^ to 10^4^ CFU/mL) to high (10^6^ to 10^7^ CFU/mL) quantities. Compared to laboratory culture-based methods, the multiplex qPCR diagnostic specificity (Sp) was 100% (95% CI [86.8–100]; *n* = 26) and diagnostic sensitivity (Se) was 92.3% (95% CI [74.9–99.1]; *n* = 26) for *Mycoplasma* species in milk samples collected from California dairy farms. Similarly, the Sp was 100% (95% CI [90.5–100]; *n* = 37) and Se was 93.3% (95% CI [68.1–99.8]; *n* = 15) for *M. bovis*. Our assay can detect and distinguish among *M. bovis*, other prevalent *Mycoplasma* spp., and non-pathogenic *Acholeplasma laidlawii* for effective identification and control of mycoplasma mastitis, ultimately supporting dairy cattle health and high-quality dairy products in California.

## Introduction

Mastitis caused by *Mycoplasma* species is highly infectious in dairy cattle and causes serious disease and economic burdens, especially in large dairy herds in the U.S. and other countries worldwide (Nicholas, Fox & Lysnyansky, 2016). Pathogenic *Mycoplasma* spp. are unique compared to most other bacterial causes of mastitis because they lack a cell wall and have smaller genomes (Fox, Kirk & Britten, 2005). These organisms infect the udder tissue of dairy cows, and the resultant disease has long-term effects on milk quality, yield, and animal health (Nicholas, Fox & Lysnyansky, 2016). In the US alone, the mean cost per clinical mastitis case caused by *Mycoplasma* spp. and other microorganisms besides Gram-positive and Gram-negative bacteria was estimated at US $95.31 annually, with majority of the costs attributed to treatment (Cha et al., 2011). However, because of the very contagious nature and recognized poor response to therapy, dairy cattle with udder infections due to *Mycoplasma* spp. are often removed from the herd following identification of infection which significantly adds to the economic burden of mycoplasma mastitis (Ball, 1999; Maunsell et al., 2011; Nicholas, Fox & Lysnyansky, 2016). The most prevalent species causing cattle mastitis is *Mycoplasma bovis*, but other *Mycoplasma* species including *Mycoplasma californicum* and *Mycoplasma bovigenitalium* are also of diagnostic interest to dairy cattle farmers in California (Kirk et al., 1997; Infante-Martinez, Aguado & Eduard-Jasper, 1999; Brenner et al., 2009).

Infectious *Mycoplasma* spp. are highly contagious, frequently resistant to antibiotic treatment, and can result in chronic and subclinical states of infection. Because *Mycoplasma* can spread quickly, mastitis caused by *Mycoplasma* spp. is particularly detrimental to large (>500 cow) dairy herds (Jasper, 1982; Pfutzner & Sachse, 1996). For these reasons, screening of bulk tank milk or pooled animal milk samples is important to facilitate removal of infected animals thereby limiting the spread of infection. Routine *Mycoplasma* investigations involve using laboratory-culture based methods to enrich the bacteria from milk. This approach is challenging considering the numerous nutrient requirements and specific incubation conditions required for *Mycoplasma* growth. Even under optimal conditions, some mycoplasma strains are still difficult to grow on standard laboratory culture media (Razin, 1996). Other challenges include the extended incubation time (approximately 7 to 10 days) required before reaching sufficient cell numbers for analysis and also the time needed to identify *Mycoplasma* at the species level, usually by serological testing. Additionally, phenotypic similarities between *Mycoplasma* spp. and commensal environmental contaminants, most predominantly, *Acholeplasma laidlawii*, can result in false positives (Pfutzner & Sachse, 1996; Clothier et al., 2010; Fox, 2012).

Nucleic-acid based, culture-independent methods are now compelling alternatives for *Mycoplasma* diagnosis because of their potential to be rapid, accurate, and cost effective. Use of PCR and qPCR-based tests, in particular, have the greatest promise to be the most rapid and reliable methods for *Mycoplasma* spp. detection, identification, and quantification. To this regard, several end-point PCR and quantitative real-time PCR (qPCR) assays for *M. bovis* have been developed targeting *M. bovis* 16S rRNA (van Kuppeveld et al., 1992; Chavez Gonzalez et al., 1995; Cornelissen et al., 2017), *uvrC* (Clothier et al., 2010; Rossetti, Frey & Pilo, 2010; Naikare et al., 2015; Gioia et al., 2016; Behera et al., 2018), *oppD* (Sachse et al., 2010), *fusA* (Boonyayatra et al., 2012), and *gltX* (Appelt et al., 2019).

When assessing outbreaks of mycoplasma mastitis in dairies, especially for California’s large dairy herds, multiplex qPCR can be a favorable and practical approach for diagnostic testing. The use of different primer and probe sets in a single reaction tube significantly shortens the detection times and increases throughput capacity (Chapela, Garrido-Maestu & Cabado, 2015). A multiplex qPCR assay was developed to detect *M. bovis*, *M. californicum*, and *M. bovigenitalium* in milk samples with a limit of detection of between 10^2^ to 10^5^ colony forming units (CFU) per mL (Parker et al., 2017). There are also commercially-available, multiplex qPCR kits for milk such as PathoProof, Mastitis Major-3 kit (Thermo Fisher Scientific, Finland), and *bactotype* Mastits HP3 PCR Kit (Qiagen, Leipzig, Germany) designed to detect *M. bovis* along with the other contagious mastitis pathogens *Staphylococcus aureus* and *Streptococcus agalactiae*. However, compared to *Mycoplasma* spp., both *S. aureus* and *S. agalactiae* are relatively easy to isolate using conventional aerobic culture methods with final results available within 48 h.

In this study, we developed a multiplex qPCR assay with Taqman probes for detection of *Mycoplasma* spp., *M. bovis*, and *A. laidlawii*. The specificity and quantification range were verified for the individual primer sets as well as in multiplex. The multiplex assay was then tested for its diagnostic capability compared to routine laboratory, culture-based methods.

## Materials and Methods

### Bacterial strains and milk samples

*M. bovis* ATCC 25523, *M. californicum* ATCC 33461, *M. bovigenitalium* ATCC 19852, *Mycoplasma canadense* ATCC 29410, *Mycoplasma arginine* ATCC 23838, *Mycoplasma alkalescens* ATCC 29103 and *A. laidlawii* ATCC 23206 were purchased from the American Type Culture Collection (ATCC, Manassas, VA, USA) (Table 1). *M. bovis* ATCC 25523 and *A. laidlawii* ATCC 23206 genomic DNA (gDNA) was also purchased from the ATCC.

**Table 1:** Bacterial and algal strains used in this study.

Organism	Strain/Source^a^
*Mycoplasma bovis* *Mycoplasma californicum*	ATCC 25523 and field isolates (*n* = 3) ATCC 33461 and field isolates (*n* = 3)
*Mycoplasma bovigenitalium*	ATCC 19852 and field isolates (*n* = 3)
*Mycoplasma canadense*	ATCC 29410 and field isolates (*n* = 3)
*Mycoplasma arginini*	ATCC 23838 and field isolates (*n* = 3)
*Mycoplasma alkalescens*	ATCC 29103 and field isolates (*n* = 3)
*Acholeplasma laidlawii*	ATCC 23206 and field isolate (*n* = 3)
*Aerococcus viridans*	ATCC 700406
*Bacillus cereus*	MQ 17
*Enterobacter cloacae* subsp *cloacae*	ATCC 13847
*Enterobacter* sp.	MQ 14M0100-3
*Enterococccus faecalis*	ATCC 29212
*Enterococcus durans*	MQ 15M0470-1
*Enterococcus faecium*	ATCC 35667
*Enterococcus gallinarium*	ATCC 700425
*Escherichia coli*	ATCC 25922
*Klebsiella pneumoniae*	MQ 14
*Proteus hauseri*	ATCC 13315
*Prototheca species*	MQ 16M1258-4
*Pseudomonas aeruginosa*	ATCC 10145
*Serratia marcescens*	MQ 15M0394-3
*Staphylococcus aureus*	MQ 16M1264-9; ATCC 25923
*Staphylococcus capitis* subsp *capitis*	ATCC 35661
*Staphylococcus chromogenes*	DFSL 8435
*Staphylococcus cohnii*	ATCC 35662
*Staphylococcus epidermidis*	DFSL 1780
*Staphylococcus haemolyticus*	DFSL 8043
*Staphylococcus pasteuri*	DFSL 8109
*Staphylococcus warneri*	MQ 15M0945
*Staphylococcus xylosus*	ATCC 35663
*Streptococcus agalactiae*	ATCC 27956
*Streptococcus dysgalactiae* subsp *dysgalactiae*	ATCC 27957
*Streptococcus dysgalactiae* subsp *equisimilis*	ATCC 35666
*Streptococcus equi* subsp *zooepidemicus*	ATCC 700400
*Streptococcus equinus*	MQ 15M0757-12
*Streptococcus infantarius* subsp *coli*	ATCC 27960
*Streptococcus mutans*	MQ 15M1463-3
*Streptococcus uberis*	MQ 16M1263-101
*Streptococcus uberis*	ATCC 27958
*Streptococcus uberis*	ATCC 700407

**Note:**

a ATCC, American Type Culture Collection (Manassas, VA, USA); MQ, Milk Quality Laboratory (VMTRC; UC Davis, Tulare, CA, USA); DFSL, Dairy Food Safety Laboratory (VMTRC; UC Davis, Tulare, CA, USA).

Field isolates of *Mycoplasma* and *Acholeplasma* were obtained by routine bacteriological testing of milk samples submitted by producers and milk processors to the Milk Quality Laboratory (MQL) of the University of California, Davis Veterinary Medicine Teaching and Research Center (VMTRC, Tulare, CA, USA). The samples were from individual cows and bulk tanks located at dairies in the Central Valley, California (Table 1). All *Mycoplasma* and *Acholeplasma* isolates were recovered using VMTRC MQL protocols adapted from the National Mastitis Council (US, 2004). Briefly, milk samples were plated on modified mycoplasma agar (University of California Davis Veterinary Medicine Biological Media Services) directly upon receipt as well as after enrichment in mycoplasma specific broth (UC Davis VM Biological Media Services) for 48 h in 4% CO_2_ at 37 °C. Putative *Mycoplasma* and *Acholeplasma* colonies were streaked for isolation and subsequently identified to the species level by staining with fluorescent antibodies according to previously described methods (Baas & Jasper, 1972). To discriminate between *A. laidlawii* and *Mycoplasma* spp., a digitonin disk inhibition test was performed according to the method previously described (Thurmond, Holmberg & Luiz, 1989). All isolates were stored in 15% (v/v) glycerol at −80 °C.

Additionally, a total of 33 isolates from 30 different microbial species other than mycoplasmas and commonly found in bovine milk were used for cross-specificity evaluation of the qPCR assay (Table 1). These isolates were either obtained from the ATCC as type strains or isolated at the MQL and Dairy Food Safety Laboratory (DFSL) of University of California, Davis Veterinary Medicine Teaching and Research Center (VMTRC, Tulare, CA, USA) from routine diagnostic milk sample submissions from dairies located in the Central Valley, CA. Species identity was confirmed by 16S rRNA gene and *rpoB* sequencing (Drancourt et al., 2000; Drancourt & Raoult, 2002).

### Genomic DNA extraction

The DNeasy Blood and Tissue Lysis kit (Qiagen, Carlsbad, CA, USA) was used for bacterial gDNA extraction and purification according to the manufacturer’s instructions. The gDNA was extracted from *Mycoplasma* and *Acholeplasma* strains after incubation in mycoplasma specific broth (UC Davis VM Biological Media Services) at 37 °C in 4% CO_2_ for between 24 to 72 h (Appelt et al., 2019). The gDNA from other microbial species (Table 1) was isolated from colonies retrieved from bovine blood agar plates (UC Davis VM Biological Media Services) after incubation at 37 °C for 24 to 48 h. The commercial kit was used for the isolation of gDNA in order to support the elimination of PCR inhibitors that affect the sensitivity of the assay or even lead to false negative results (Schrader et al., 2012). For gDNA extractions from bacteria contained in milk, 1 ml milk was centrifuged at 18,407×*g* for 10 min. The resulting pellet was then suspended in Buffer ATL (Qiagen, Carlsbad, CA, USA) and Proteinase K (Qiagen, Carlsbad, CA, USA) for incubation at 56 °C for 1 h with continuous mixing prior to DNA extraction and purification. The gDNA concentrations were measured on a Qubit 3.0 Fluorometer using the Qubit dsDNA HS Assay Kit (Life Technologies, Eugene, OR, USA). All gDNA was stored at −20 °C until analysis or at 4 °C for no longer than 2 to 4 weeks.

### qPCR primer design

Clustal W (Thompson, Higgins & Gibson, 1994) was used to identify conserved and variable *Mycoplasma* and *Acholeplasma* DNA targets for the development of new primer-probe pairs to be used for the qPCR assays. DNA sequence alignments were performed for 16S rRNA, *rpoB* and intergenic transcribed spacer (ITS) region between 16S and 23S rRNA genes in *M. bovis, M. californicum, M. bovigenitalium, M. canadense M. alkalescens*, and *A. laidlawii*. Those *Mycoplasma* species were selected for primer development because of their prevalence in *Mycoplasma* mastitis reported in the US dairy herds (Fox, 2012; Nicholas, Fox & Lysnyansky, 2016) and by the VMTRC MQL. The following strains were used for primer selection: *M. bovis* strain PG45 (ATCC 25523, NC_014760) (Wise et al., 2011), *M. californium* strain ST-6 (ATCC 33461, NZ_CP007521) (Calcutt, Foecking & Fox, 2014), *M. bovigenitalium* strain HAZ (ATCC 19852, AP017902) (Edward & Freundt, 1956), *M. canadense* strain HAZ 360_1 (ATCC 29410, NZ_AP014631), *M. alkalescens* 14918 (ATCC 29103, NZ_AMWK01000000), and *A. laidlawii* PG-8A (ATCC 23206, NC_010163) (Lazarev et al., 2011). DNA sequence alignments showed sufficient nucleotide variation in the 16S rRNA and *rpoB* genes and ITS region for primer and Taqman probe design (Fig. S1).

All qPCR primers and Taqman probes were designed using Primer-BLAST and Primer Express software (Applied Biosystems, Waltham, MA, USA) (Table 2 and Fig. S1). Primers were regarded to be acceptable if they contained at least two total mismatches to unintended targets, including at least two mismatches within the last five base pairs of the 3′ end. All primers were designed to have a melting temperature at 59 °C to 62 °C and an amplicon size between 108 to 232 bp.

**Table 2:** Sequences of primers, probes, PCR product sizes and amplification efficiency of the qPCR assays^a^.

Organism	Target	Primer and probe^b^	Primer and probe sequence (5′ to 3′)	nM	Product (bp)	qPCR efficiency (%)^c^	
						Singleplex	Multiplex
*Mycoplasma* spp^d^	16S rRNA	Myco_F Myco_R Myco_Probe	CGAGCGCAACCCTTATCCTT CCCCACTCGTAAGAGGCATGA VIC-TCGTCCCCACCTTCCTCCCG-QSY	100 100 75	118	88.8–100.9	96.8–102.4
*M. bovis*	*rpoB*	M.bovis_F M.bovis_R M.bovis_Probe	TTTCAGCCGCTAACTTCAGAGC GCAAGTTCCCCATCCTTGAAG ABY-TCGCCTTTAGCAACTTCTTGACCAA-QSY	200 200 200	232	87.1	95.6
*A. laidlawii*	ITS	Achol_F Achol_R Achol_Probe	AAGTGGGCAATACCCAACGC ACGTTCCCGTAGGGATACCTTG 6-FAM-ACGGCTCCCTCCCTTTCGGG-QSY	15015075	108	91.9	91.2

**Notes:**

a The QuantiFast Multiplex PCR master mix (Qiagen, Redwood City, CA, USA) was used for all assays and with an annealing temperature of 58 °C.

b F and R indicate forward and reverse primers, respectively. TaqMan probes were designed with 6-FAM (6-carboxyfluorescein) VIC (2’-chloro-7phenyl-1,4-dichloro-6-carboxy-fluorescein) and ABY (Thermo Fisher, Waltham, MA, USA) as reporter dyes on the 5′ end and QSY 7 (QSY7 succinimidyl ester) as the quencher dye on the 3′ end.

c Amplification efficiencies were determined using gDNA from *M. bovis* ATCC25523, *M. californicum* ATCC 33461, and *M. bovigenitalium* ATCC19852 (16S rRNA), *M. bovis* ATCC25523 (*rpoB*), and *A. laidlawii* ATCC 23206 (ITS).

d The PCR amplification efficiencies for *M. bovis*, *M. californicum* ATCC 33461, and *M. bovigenitalium* ATCC19852 were measured as 88.8%, 97.1%, and 100.9% in singleplex and 96.8, 102.4% and 97.9% in multiplex, respectively.

### qPCR assay parameters

All primers and Taqman probes were optimized by testing concentrations in the range of 100 nM to 400 nM and 75 nM to 400 nM, respectively. qPCR amplification was performed in 96-well plates on an Applied Biosystems 7500 Fast thermal cycler (Applied Biosystems, Carlsbad, CA, USA) with an initial denaturation step of 5 min at 95 °C followed by 40 cycles of 45 s at 95 °C for DNA denaturation and 45 s at 58 °C for primer and probe hybridization and DNA elongation steps. The multiplex assay, combining the three individual assays for *Mycoplasma*, *M. bovis*, and *A. laidlawii*, was carried out in a total volume of 20 µl, comprising 5 µl of the gDNA, 10 µl of 2X QuantiFast Multiplex PCR master mix (Qiagen, Redwood City, CA, USA), and between 75 and 200 nM of each primer and TaqMan probe (Table 2). qPCR assay efficiencies were calculated using standard curves based on 10-fold serial dilutions of gDNA from *M. bovis* ATCC 25523 for the *rpoB* and 16S rRNA assays and *A. laidlawii* ATCC 23206 for the ITS assay.

### Quantitative ranges of the qPCR assays

Serial dilutions were prepared of gDNA from *M. bovis* ATCC 25523, *M. californicum* ATCC 33461, *M. bovigenitalium* ATCC 19852, and *A. laidlawii* ATCC 23206. The serial dilutions spanned a 10^6^-fold range, encompassing between approximately 5 fg to 5 ng gDNA. The QuantiFast Multiplex PCR Master mix was used in combination with the primer and Taqman probe concentrations described in Table 2 with an annealing temperature of 58 °C. The assays were tested in singleplex and multiplex in triplicate and in two separate runs and for each gDNA primer/probe combination. To estimate genome equivalents, it was assumed that the average base pair weighs 650 g/mol and the average genome sizes were 1 Mbp for *M. bovis* (Wise et al., 2011), 0.79 Mbp for *M. californicum* (Calcutt, Foecking & Fox, 2014), 0.86 Mbp for *M. bovigenitalium* (Hata, Nagai & Murakami, 2017), and 1.5 Mbp for *A. laidlawii* (Lazarev et al., 2011). For verifying assay specificity, a total of 1 to 25 ng gDNA from diverse microbial strains (Table 1) was tested for detection.

To examine the quantitative range of the multiplex assay in milk, raw bovine milk samples were first confirmed to be free of *Mycoplasma* spp. by plating on modified mycoplasma agar and incubation at 37 °C in 4% CO_2_ for 7 days. Next, 1 × 10^6^ to 1 × 10^8^ colony-forming units (CFU)/mL of *M. bovis* ATCC 25523, *M. californicum* ATCC 33461, and *A. laidlawii* ATCC 23206 were inoculated into separate aliquots of that milk. The cell suspensions were immediately used for serial dilutions in the same raw milk so that the final cell numbers spanned a 10^4^-fold (*M. californicum*) to 10^6^-fold (*M. bovis* and *A. laidlawii*) range. gDNA was then extracted and tested with the multiplex assay using conditions described in Table 2 before setting multiplex qPCR assay detection criteria.

**Table 3:** Criteria for detection of *Mycoplasma* spp., *M. bovis, and A. laidlawii* in milk with the multiplex qPCR assay.

Organism^b^	Gene target (Cut-off *Ct* value)^a^		
	16S rRNA (*C*_*t*_ ≤ 32)	*rpoB* (*C*_*t*_ ≤ 33)	ITS (*C*_*t*_ ≤ 32)
*Mycoplasma* spp.	+	–	–
*M. bovis* ^c^	+	+	–
*A. laidlawii*	–	–	+

**Notes:**

a *Ct*, Threshold Cycle value; positive (+) and negative (−) symbols indicate a positive and negative result for that group of bacteria by the multiplex qPCR assay.

b *M. californicum* ATCC33461 *M. bovis* ATCC25523, and *A. laidlawii* ATCC23206 were used for determining LOD levels of *Mycoplasma, M. bovis*, and *A. laidlawii*, respectively.

c *M. bovis* gDNA was expected to be detected by both the 16S rRNA and *rpoB* assays, therefore a positive *M. bovis* sample is expected to have <3 *Ct* value difference for those targets.

### Multi-species detection

Mixtures of *M. bovis* ATCC 25523 and *A. laidlawii* ATCC 23206 gDNA were tested with the multiplex assay to assess for the capacity for simultaneous, multi-species detection. gDNA was mixed instead of intact cells because growth rate variations between the strains limited our capacity to obtain sufficient cell numbers within the same period of time. To prepare the gDNA mixtures, gDNA was extracted from a single strain immediately after inoculation into separate aliquots of raw milk at either low (10^2^ to 10^3^ CFU/mL), medium (10^3^ to 10^4^ CFU/mL), or high (10^6^ to 10^7^ CFU/mL) cell numbers. Next, gDNAs from those strains were mixed in equal volumes (1:1 ratios) and tested with the multiplex assay using the assay conditions described in Table 2. A total of nine combinations of gDNAs, representing low, medium, and high levels of *M. bovis* and *A. laidlawii* cells were measured.

**Table 4:** **Detection of *M. bovis* and *A. laidlawii* mixtures in milk with the multiplex qPCR assay**^a^.

		Target	Ct (avg ± stdev)^b^
***M. bovis*** (low)	*A. laidlawii* (low)	16S rRNA	33.47 ± 0.24
	*A. laidlawii* (intermediate)		33.32 ± 0.12
	*A. laidlawii* (high)		33.41 ± 0.41
	*A. laidlawii* (low)	*rpoB*	33.80 ± 0.52
	*A. laidlawii* (intermediate)		33.47 ± 0.21
	*A. laidlawii* (high)		33.49 ± 0.66
	*A. laidlawii* (low)	ITS	32.91 ± 0.38
	*A. laidlawii* (intermediate)		25.65 ± 0.07
	*A. laidlawii* (high)		15.79 ± 0.11
***M. bovis*** (intermediate)	*A. laidlawii* (low)	16S rRNA	27.44 ± 0.11
	*A. laidlawii* (intermediate)		27.42 ± 0.12
	*A. laidlawii* (high)		27.22 ± 0.16
	*A. laidlawii* (low)	*rpoB*	27.05 ± 0.23
	*A. laidlawii* (intermediate)		26.97 ± 0.10
	*A. laidlawii* (high)		26.83 ± 0.31
	*A. laidlawii* (low)	ITS	32.87 ± 0.51
	*A. laidlawii* (intermediate)		25.71 ± 0.07
	*A. laidlawii* (high)		15.77 ± 0.07
***M. bovis*** (high)	*A. laidlawii* (low)	16S rRNA	16.68 ± 0.15
	*A. laidlawii* (intermediate)		16.63 ± 0.18
	*A. laidlawii* (high)		16.90 ± 0.05
	*A. laidlawii* (low)	*rpoB*	16.38 ± 0.19
	*A. laidlawii* (intermediate)		16.42 ± 0.22
	*A. laidlawii* (high)		16.58 ± 0.14
	*A. laidlawii* (low)	ITS	36.70 ± 0.71
	*A. laidlawii* (intermediate)		26.02 ± 0.15
	*A. laidlawii* (high)		15.69 ± 0.05

**Notes:**

a gDNA was extracted from milk inoculated with *M. bovis* ATCC 25523 and *A. laidlawii* ATCC 23206 at either low (10^2^ to 10^3^ CFU/mL), medium (10^3^ to 10^4^ CFU/mL), or high (10^6^ to 10^7^ CFU/mL).

b Shading indicates average *Ct* values within range for detection (*n* = 3) using qPCR criteria for multiplex assay given in Table 3.

### qPCR performance on field milk samples

To compare the qPCR assay performance to conventional culture-based methods, a total of 52 frozen (−20 °C) bulk tank and individual cow milk samples were first confirmed to be either *Mycoplasma* spp. culture positive or negative. This was determined by plating the milk samples directly on modified mycoplasma agar as well as after enrichment in mycoplasma specific broth for 24 h in 4% CO_2_ at 37 °C. Colonies were evaluated by the same person at 4 and 7 days and verified as *M. bovis, M. bovigenitalium*, or *M. alkalescens* using staining with fluorescent antibodies according to previously described methods (Baas & Jasper, 1972). The 52 milk samples were then anonymized for testing by the multiplex qPCR assay and 5 µl of total DNA extracted from the milk was tested using assay parameters described in Table 2. Diagnostic sensitivity (Se) and diagnostic specificity (Sp) of the multiplex qPCR assay was calculated as described previously (Gioia et al., 2016). Using the results from laboratory culture as the reference, the number of true positive (TP), true negative (TN), false positive (FP), and false negative (FP) samples were combined into following formulas: Se (%) = TP/(TP + FN) and Sp (%) = TN/(TN + FP). Kappa, an estimate of agreement beyond chance between laboratory culture and qPCR was estimated and interpreted as <40%, poor; 41 to 75%, fair to good; >75%, excellent (Fleiss, Levin & Paik, 2003).

## Results

### qPCR assay efficiency and range of detection for target gDNA

**Figure 1:**
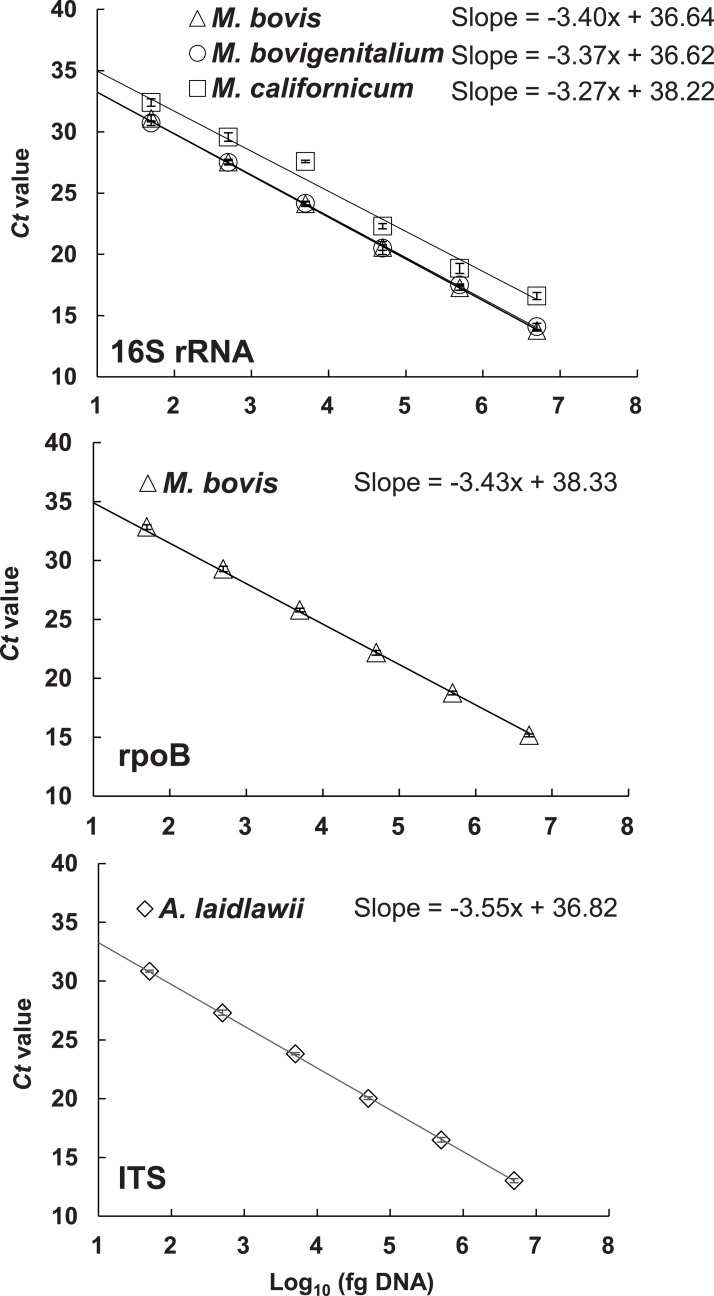
Standard curves for the 16S rRNA, *rpoB* and ITS TaqMan qPCR assays performed in multiplex. The standard curves were constructed with 10-fold serial dilutions of *M. bovis* ATCC 25523 (16S rRNA and rpoB assays), *M. bovigenitalium* ATCC 19852 (16S rRNA assay), *M. californicum* ATCC 33461 (16S rRNA assay), and *A. laidlawii* ATCC 23206 (ITS assay) gDNA, ranging from between approximately 5 fg to 5 ng gDNA. Results shown are from a single run with each dilution tested in triplicate. The R^2^ value was 0.99 for the standard curve of each target (16S rRNA, *rpoB* and ITS). Error bars indicate standard deviation (±) based on the results for three replicates.

The *Mycoplasma* 16S ribosomal RNA (16S rRNA) gene, *M. bovis rpoB*, and the *A. laidlawii* ITS region were selected for qPCR primer design. These targets were previously shown to be useful markers for detection of *Mycoplasma* and *Acholeplasma* in milk and clinical samples (van Kuppeveld et al., 1992; Cornelissen et al., 2017; Gioia et al., 2016; Parker et al., 2017; Tang et al., 2000). After testing a range of concentrations for each primer and TaqMan probe, the Taqman qPCR assay parameters (QuantiFast Multiplex PCR master mix; annealing temperature of 58 °C) and assay primer and probe concentrations were set as described in Table 2. The qPCR amplification efficiencies for each of the individual qPCR assays on intended target gDNA ranged from 90% to 102.4% when tested separately (in singleplex) (Table 2 and Fig. S2) and combined into one *Mycoplasma-M. bovis-A. laidlawii* multiplex assay (Table 2 and Fig. 1). For the 16S rRNA gene assay in multiplex, the qPCR amplification efficiency was high for *M. bovis* (Table 2) (96.8%) and comparable to efficiencies found for *M. californicum* ATCC 33461 (102.4%) and *M. bovigenitalium* ATCC19852 (97.9%). The correlation coefficient of the straight line, *R*^*2*^ was higher than 0.98 for all assays over a 10^6^-fold range in singleplex (Fig. S2) and multiplex (Fig. 1). The lower limit of detection (LLD) for each assay was similar between the singleplex (Fig. S2) and multiplex (Fig. 1) formats (*Ct* values from 30 to 35). Both formats were able to detect low numbers of DNA copies per PCR (50 fg DNA, approximately equal to 30 to 58 genome equivalents for the respective species being targeted by the assay).

### Specificity of the multiplex qPCR assay

We next examined the qPCR assays for cross-reactivity between the other species targets contained in the *Mycoplasma-M. bovis-A. laidlawii* multiplex test. No amplification (*Ct* > 37) was found for the *A. laidlawii* 16-23S ITS assay when tested on either *M. bovis* ATCC25523, *M. californicum* ATCC33461, or *M. bovigenitalium* ATCC19852 gDNA template (data not shown). For the *M. bovis rpoB* assay, some amplification (*C*_*t*_ values 31 to 37) was observed for *M. californicum* ATCC 33461 and *M. bovigenitalium* ATCC 19852 gDNA. However, that *Ct* range of detection was approximately 17 *Ct* values higher than observed for equivalent quantities of the *M. bovis* target (0.5 ng), comparable to a 10^5^ to 10^6^-fold difference in detection sensitivity. For the *Mycoplasma* 16S rRNA assay, some amplification (*C*_*t*_ values 31 to 35) was observed for *A. laidlawii* ATCC 23206 gDNA at the highest quantity of gDNA tested (0.5 ng). However, just as found for the *rpoB* assay, those *Ct* values were also much higher (*Ct* difference of 17) than when *A. laidlawii* was used as the target, comparable to a 10^5^ to 10^6^-fold difference in detection.

No amplification (*Ct* > 37) was observed with the multiplex assay for any non-*Mycoplasma* strains from 33 different microbial species, including representatives of the *Staphylococcus, Bacillus, Aerococcus, Enterococcus, Enterobacter, Escherichia, Klebsiella, Proteus, Prototheca, Pseudomonas, Serratia*, and *Streptococcus* genera (Table 1). As expected, gDNA from *M. californicum, M. bovigenitalium, M. canadense, M. arginini*, and *M. alkalescens* isolates (Table 1) resulted in amplification with the 16S rRNA assay (*Ct* values < 28) but not with the *rpoB* and ITS assays (*Ct* values > 37). Moreover, gDNA from *A. laidlawii* field isolates only resulted in amplification with the ITS assay, while three other field isolates of *M. bovis* from milk were accurately detected by the *rpoB* and 16S rRNA assays (*Ct* values ranged from between 21 to 24).

### Limit of Detection (LOD) of multiplex qPCR assay in milk

**Figure 2:**
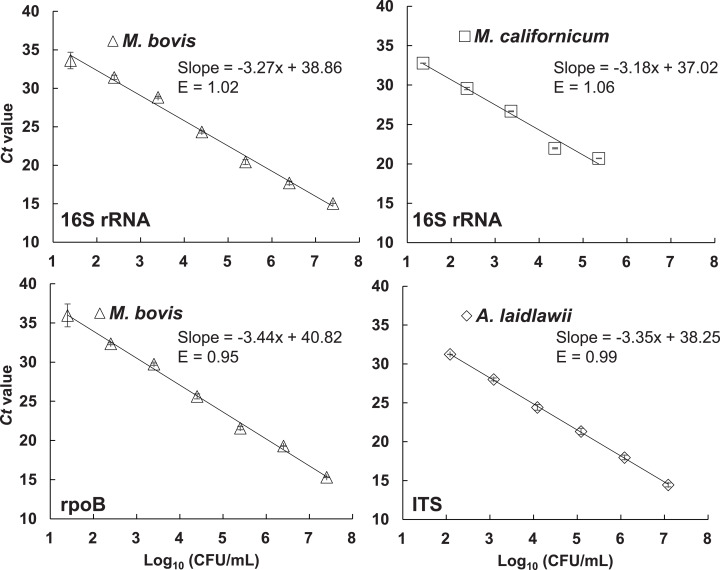
Limit of detection of the multiplex qPCR assay for *M. bovis*, *M. californicum*, and *A. laidlawii* in milk. Serial dilutions of *M. bovis* ATCC 25523 (starting cell number of 2.5 × 10^8^ CFU/mL), *M. californicum* ATCC 33461 (starting cell number of 2.3 × 10^6^ CFU/mL), and *A. laidlawii* ATCC 23206 (1.22 × 10^8^ CFU/mL) were performed in raw milk confirmed to be negative for *Mycoplasma* and *A. laidlawii* prior to gDNA extraction and target detection with the multiplex qPCR assay. Results shown are from a single run with each dilution tested in duplicate. The R^2^ value was 0.99 for the standard curve of each target (16S rRNA, *rpoB* and ITS). Error bars indicate standard deviation (±) based on the results for all replicates.

Next, the multiplex qPCR assay range of detection was tested for *Mycoplasma* and *A. laidlawii* in milk. A DNA amplification efficiency of 95% to 106% (*R*^*2*^ > 0.98) was found for all three gene targets spanning a 10^4^ to 10^6^-fold range when the multiplex assay was applied on gDNA extracted from serial dilutions of *M. californicum* ATCC33461, *M. bovis* ATCC25523, or *A. laidlawii* ATCC23206 contained in milk (Fig. 2). However, it was noted that milk alone (no-template control or mycoplasma culture negative) sometimes resulted in some DNA amplification (*Ct* cycles 33 to 37). Therefore, *Ct* values above 33 were reported as negative for the multiplex qPCR assay. To avoid false positives, a LOD cut-off *Ct* value was set at <32 for *Mycoplasma*, <33 for *M. bovis*, and <32 for *A. laidlawii* (Table 3). Those cut-off *Ct* values for each target assay were selected based on a minimum 3-Ct difference between the lowest quantities of the target organism in milk and the negative control *Ct*. Based on cell numbers resulting in that *Ct*, the LOD was within the range of 120 to 250 CFU/mL.

### Multi-species detection with the multiplex qPCR assay

Application of the multiplex qPCR assay on gDNA from *M. bovis* ATCC 25523 and *A. laidlawii* ATCC 23206 mixed in different proportions showed that multiplex assay could simultaneously detect *M. bovis* and *A. laidlawii* present in the same sample (Table 4). The 16S rRNA and *rpoB* targeted assays were positive when gDNA from milk containing either moderate (10^3^ to 10^4^ CFU/mL) to high (10^6^ to 10^7^ CFU/mL) numbers of *M. bovis* was applied, irrespective of *A. laidlawii* gDNA abundance. Similarly, the *A. laidlawii* ITS qPCR assay results were positive when gDNA from milk containing moderate or high numbers of *A. laidlawii* was used, even in the presence of high quantities of *M. bovis* gDNA. Neither organism was detectable according to qPCR when the gDNA was extracted from milk with low cell numbers (10^2^ to 10^3^ CFU/mL) (Table 4).

### qPCR performance on field milk samples

To determine the extent to which the multiplex qPCR assay can reliably detect and differentiate between *M. bovis* from non-*bovis Mycoplasma* spp. and *A laidlawii*, a total 52 milk samples collected from bulk tanks and individual cows on CA dairy farms were analyzed with the assay and the results were compared to laboratory culture-based detection methods (Table 5). No sample was shown to contain *A. laidlawii* and therefore the presence of that organism in field milk samples was not evaluated. Using the *Mycoplasma* spp. qPCR Ct value criteria we set for the multiplex assay (Table 3), no false-positive results, Sp 100% (95% CI [86.8–100]; *n* = 26) were obtained for the milk samples which were also found to lack *Mycoplasma* spp. according to laboratory culture-based methods (Table 5). Similarly, using the *M. bovis* criteria (Table 3), no false positive results, Sp 100% (95% CI [90.5–100]; *n* = 37) were obtained for the milk samples found to lack *M. bovis* according to laboratory culture-basedmethods (Table 5). Notably, the assay parameters were discriminative because even though a sample containing *M. alkalescens* (ID# 482582) yielded a Ct value of approximately 32 for the *M. bovis* qPCR assay, there was an approximate five-Ct difference between the *rpoB* and 16S rRNA assays. That difference was beyond the allowable limit for *M. bovis* by the multiplex assay, and therefore was appropriately identified as having *Mycoplasma* spp. and not *M. bovis*. (Table 3).

**Table 5:** Laboratory culture and multiplex qPCR results for field milk samples.

		Laboratory Culture^b^			Multiplex qPCR Result (*Ct*)^c^			
ID#	Sample Type^a^	DP	MB	Organism	Gene Target			Organism
					16S rRNA	*rpoB*	ITS	
4750	C	≥400	+	*M. bovis*	+(15.10)	+(17.18)	–	*M. bovis*
1331-2	T	4	+	*M. bovis*	+(29.79)	+(31.43)	–	*M. bovis*
1985-1	T	85	+	*M. bovis*	+(24.60)	+(25.90)	–	*M. bovis*
2927-3	T	2	–	*M. bovis*	+(31.03)	−(33.76)	–	*Mycoplasma* sp.
3145-1	T	+	+	*M. bovis*	+(27.70)	+(32.35)	–	*M. bovis*
3606-1	T	+	+	*M. bovis*	+(26.97)	+(28.34)	–	*M. bovis*
3642-1	T	≥100	+	*M. bovis*	+(25.16)	+(27.80)	–	*M. bovis*
3915-1	T	+	+	*M. bovis*	+(26.17)	+(27.32)	–	*M. bovis*
43722-1	T	≥200	+	*M. bovis*	+(23.19)	+(24.91)	–	*M. bovis*
43897-1	T	4	+	*M. bovis*	+(28.81)	+(31.46)	−(34.4)	*M. bovis*
4395-1	T	≥600	+	*M. bovis*	+(26.27)	+(27.55)	–	*M. bovis*
43967-2	T	1	+	*M. bovis*	+(29.42)	+(31.45)	−(34.52)	*M. bovis*
4435-1	T	9	+	*M. bovis*	+(29.44)	+(30.62)	−(36.74)	*M. bovis*
4437-1	T	≥200	+	*M. bovis*	+(23.94)	+(24.95)	−(35.99)	*M. bovis*
4784-1	T	9	+	*M. bovis*	+(27.63)	+(29.77)	−(34.96)	*M. bovis*
8229	C	–	+	*M. bovigenitalium*	+(17.84)	−(34.89)	−(37.21)	*Mycoplasma sp*.
180	C	–	+	*M. alkalescens*	+(31.16)	–	–	*Mycoplasma sp*.
6141	C	–	+	*M. alkalescens*	+(29.31)	−(38.47)	–	*Mycoplasma sp*.
33257	C	–	+	*M. alkalescens*	+(26.78)	−(35.35)	–	*Mycoplasma sp*.
39494	C	–	+	*M. alkalescens*	+(25.85)	−(38.41)	–	*Mycoplasma sp*.
41672	C	–	+	*M. alkalescens*	–	–	–	–
42275	C	6	+	*M. alkalescens*	+(25.25)	−(36.52)	−(34.61)	*Mycoplasma sp*.
43960	C	–	+	*M. alkalescens*	−(34.67)	–	−(32.43)	–
482582	C	1	+	*M. alkalescens*	+(26.46)	−(31.54)	–	*Mycoplasma sp*.
749	C	85	+	*M. alkalescens*	+(19.99)	−(35.69)	–	*Mycoplasma sp*.
750	C	–	+	*M. alkalescens*	+(26.76)	−(34.34)	–	*Mycoplasma sp*.
829	C	–	–	–	−(32.42)	−(34.28)	−(34.89)	–
818	C	–	–	–	−(33.07)	−(35.25)	−(35.56)	–
802	C	–	–	–	−(33.18)	−(33.88)	–	–
828	C	–	–	–	−(33.43)	−(34.23)	−(34.07)	–
809	C	–	–	–	−(33.57)	−(34.22)	–	–
819	C	–	–	–	−(34.19)	−(34.09)	−(34.71)	–
815	C	–	–	–	−(34.54)	–	–	–
810	C	–	–	–	−(34.57)	−(34.58)	–	–
826	C	–	–	–	−(34.83)	−(34.35)	−(36.11)	–
823	C	–	–	–	−(34.96)	–	−(33.46)	–
804	C	–	–	–	−(35.26)	–	−(36.09)	–
822	C	–	–	–	−(35.40)	–	–	–
814	C	–	–	–	−(35.85)	–	–	–
824	C	–	–	–	−(36.02)	−(38.96)	−(35.90)	–
801	C	–	–	–	−(36.03)	–	–	–
808	C	–	–	–	−(36.06)	−(34.92)	−(35.19)	–
803	C	–	–	–	−(36.17)	−(39.70)	–	–
820	C	–	–	–	−(36.94)	–	−(34.57)	–
812	C	–	–	–	−(37.08)	−(34.66)	–	–
817	C	–	–	–	−(37.37)	–	–	–
806	C	–	–	–	−(37.65)	−(35.19)	−(34.94)	–
811	C	–	–	–	−(37.90)	–	–	–
813	C	–	–	–	−(38.00)	−(35.11)	−(34.94)	–
821	C	–	–	–	−(38.25)	–	–	–
807	C	–	–	–	–	–	−(34.88)	–
816	C	–	–	–	–	–	−(34.84)	–

**Notes:**

a Cow (C) or Bulk Tank (T) milk sample.

b DP, Direct plating. Based on results from direct plating of milk samples on modified mycoplasma agar using cotton swabs; MB, Mycoplasma Broth. Based on culture results of 24 h enriched broth samples; Plates with bacterial growth are indicated by a plus (+) and no growth by a minus (−); *Mycoplasma* spp. were identified by fluorescently labeled, species-specific antibody staining method. The organism column is shaded gray to show the species identified by laboratory culture based methods.

c The positive qPCR results are indicated by a plus (+) and negative results by a minus (−); *Mycoplasma* spp. were identified by *Ct* cut-off criteria (Table 3). The organism column is shaded gray to show the species was identified by the multiplex qPCR assay.

A total of 24 out of 26 milk samples that tested positive for *Mycoplasma* spp. according to CFU isolation in laboratory culture medium were also positive according to qPCR, Se 92.3% (95% CI [74.9–99.1]; *n* = 26). The remaining two samples (ID# 41672 and ID# 43960) were negative by qPCR for all three gene targets but tested positive for *M. alkalescens* after enrichment and plating for CFU identification (Table 5).

The multiplex assay also accurately detected 14 out of 15 milk samples determined to contain *M. bovis* according to culture-based methods, yielding a Se of 93.3% (95% CI [68.1–99.8]; *n* = 15) for *M. bovis*. The remaining milk sample (ID# 2927-3) tested positive for *Mycoplasma* spp. but not *M. bovis* (Table 5). That sample resulted in only two presumptive *M. bovis* colonies (approximately 100 CFU/ml) (Table 5). Notably the average *Ct* for that sample (*Ct* of 33.76) was only slightly above the upper *Ct* value cut-off for *M. bovis* (*Ct* ≤ 33) (Table 3) and the *Ct* for *Mycoplasma* detection with the 16S rRNA gene was near to the limit of the acceptable range (*Ct* of 31.03 compared to *Ct* < 32).

Both assays, laboratory culture and qPCR, had excellent agreement beyond chance as measured by Kappa. Specifically, the agreement between laboratory culture and qPCR in detecting milk samples testing positive for *Mycoplasma* spp. was 92.31% (SE 13.83; *P* value < 0.01) and for *M. bovis* was 95.22% (SE 13.85; *P* value < 0.01).

## Discussion

The TaqMan multiplex qPCR assay developed here was shown to be a rapid and reliable diagnostic tool for detection of *Mycoplasma* spp. and *M. bovis* and discrimination between *Mycoplasma* and *A. laidlawii* in milk. The assay was accurate and showed a large dynamic range for quantification (10^6^ fold range) while maintaining a comparable sensitivity to that of the individual qPCR assays for each target. Simultaneous monitoring was achieved for *M. bovis* and *A. laidlawii* and the diagnostic sensitivity and field performance of the multiplex qPCR assay was confirmed. The assay is a significant advancement for mastitis diagnostics because it emphasizes *M. bovis* detection while also affording the possibility to monitor for other *Mycoplasma* genera and simultaneous differentiation of non-pathogenic *A. laidlawii* contaminants.

The individual qPCR assays used here target genes that have also previously shown to be useful for *Mycoplasma* and *A. laidlawii* sensing by other methods. Conventional PCR assays targeting *Mycoplasma* 16S rRNA genes were used for detection (Hirose et al., 2001) and both detection and differentiation of different *Mycoplasma* spp., including *M. bovigenitalium* and *M. californicum* (McAuliffe et al., 2005). For developing genus- specific primers, target sequences used should have the greatest sequence divergence between genera but be conserved for species within the same genus. The 16S rRNA gene is an ideal target in this regard since the presence of two gene copies also increases the detection limit by PCR. In comparisons between the 16S rRNA genes of *Mycoplasma* and other related species including *M. californicum*, *M. bovigenitalium*, *M. alkalescens*, *M. arginini* and *M. canadense*, we observed significant sequence similarity in the V7 variable region. This finding agreed with a previous study which concluded that mycoplasma genus-specific sequences occur between the 16S rRNA V6 and V7 regions, although some cross reactivity was found with *Acholeplasma* species (van Kuppeveld et al., 1992). The alignment of the V7 regions showed a 118-bp sequence which distinguished *A. laidlawii* from *Mycoplasma* spp., while was also conserved among *Mycoplasma* species.

A variety of *M. bovis* genes including, *uvrC, gltX, fusA* and *oppD* have been targets for species detection by PCR methods (Clothier et al., 2010; Rossetti, Frey & Pilo, 2010; Sachse et al., 2010; Boonyayatra et al., 2012; Naikare et al., 2015; Gioia et al., 2016; Behera et al., 2018; Appelt et al., 2019). We selected *rpoB*, the gene encoding the highly conserved beta-subunit of the bacterial RNA polymerase, for assay development because there were more nucleotide variations in this gene between *M. bovis* and other *Mycoplasma* species. *rpoB* was also found to be a suitable target for phylogenetic analysis of *Mycoplasma* spp. including *M. bovis* (Kim et al., 2003). The use of the *rpoB* gene for *M. californicum* detection in milk has already been explored in a probe-based, qPCR assay (Boonyayatra et al., 2012; Parker et al., 2017). Therefore, in this study, we used a 232-bp sequence in *rpoB* gene for the design of primers and a Taqman probe that could detect *M. bovis*. Notably, this assay reached a higher amplification efficiency than the qPCR assay we previously developed for *M. bovis* targeting *gltX* (Appelt et al., 2019).

For the detection of *A. laidlawii*, the ITS region (108-bp; separating the two 16S–23S rRNA operons in *A. laidlawii*) was used for species-specific primers and Taqman probe development. The ITS region was previously shown to have sufficient heterogeneity to differentiate between *Mycoplasma* spp. and *A. laidlawii* (Tang et al., 2000; Gioia et al., 2016). This finding is consistent with the lack of amplification observed for *M. bovis* or the other *Mycoplasma* tested with that assay.

The individual qPCR assays performed well when combined in multiplex qPCR, with no observed cross-reactivity between primer pairs and impacts on the dynamic range of target detection. The multiplex assay was also capable of measuring gDNA from as few as 210 to 250 CFU/mL in milk for each of the targets. The sensitivity of this qPCR method is comparable to other assays which reported a detection limit for *M. bovis* in a range of between 10 and 240 CFU/mL in milk (Clothier et al., 2010; Sachse et al., 2010; Boonyayatra et al., 2012). Our assay was also more sensitive than the LOD reported for a multiplex qPCR assay targeting *uvrC* in *M. bovis*, *rpoB* in *M. californicum*, and the ITS region in *M. bovigenitalium* (Parker et al., 2017). In that study, the detection limit was 130 CFU/mL, 600 CFU/mL, and 10^5^ CFU/mL for *M. bovis*, *M californicum*, and *M. bovigenitalium*, respectively. Sensitivity decreased by 100- to 1,000-fold when all three species were present at the same time to simulate a multispecies infection (Parker et al., 2017). Our prior qPCR assay and methods targeting the *M. bovis gltX* was less sensitive to detect low quantities of *M. bovis* cells. The detection limit was between 10 to 100 gDNA copies per reaction, corresponding to 10^4^ to 10^5^ cells per mL (Appelt et al., 2019). However, it should be noted that a rapid DNA extraction method was used along with other Taq polymerase reagents which may have impacted estimates of *M. bovis* detection limits.

Recently, a survey study performed on 95 New York dairy farms detected both *M. bovis* and *A. laidlawii* in the same sample in 6% of the farms and mixed mycoplasma infections in 14% of the farms (Gioia et al., 2016). Multispecies detection could be useful when testing for mastitis because the occurrence of mixed mycoplasma spp. infection has been reported previously, with *M. bovis* being isolated in approximately 90% of those cases and other species of mycoplasma isolated as secondary pathogens or contaminants (Kirk et al., 1997; Szacawa et al., 2015). Our multiplex qPCR assay reliably detected *M. bovis* and *A. laidlawii* gDNA when mixed together. This indicated that *M. bovis* would be detectable, even with high levels of *A. laidlawii* contaminants. Future studies will investigate the sensitivity of the multiplex qPCR assay for mixed *Mycoplasma* species present in milk samples.

The multiplex assay showed a high level of Sp and Se when tested on milk samples collected from different dairy farms in CA. Multiplex qPCR yielded a Se of 92.3% for 16S rRNA and 93.3% for *rpoB* and a Sp of 100% for each of the targets compared with milk samples confirmed by traditional laboratory bacterial culture methods. These results are comparable to those obtained by others examining for *M. bovis* in milk (Clothier et al., 2010; Parker et al., 2017; Appelt et al., 2019). Even though the sample size for the field validation test was small, no false positive results were obtained. Instead, such samples were correctly identified as negatives by qPCR as they did not meet the assay criteria for species/genera identification even when culture results were positive. We suggest that qPCR negative results can be associated with either the presence of PCR inhibitors or low *Mycoplasma* cell numbers which might have led to a general decrease in PCR sensitivity. In the former case the utilization of a DNA internal control in the same tube as the target can eliminate false-negative PCR results caused by PCR inhibiting substances. In the latter case, the multiplex assay cannot be used for direct detection in milk samples containing bacterial numbers below the detection limit as it requires those samples to be enriched before detection. Estimation of the diagnostic sensitivity and specificity for *A. laidlawii* was not possible given that none of the study samples tested positive for *A. laidlawii* by laboratory culture. In addition, estimates of diagnostic accuracy for *Mycoplasma* spp. and *Mycoplasma bovis* reported here should be interpreted with caution given the small sample size used in their diagnostic test evaluation.

## Conclusions

Because the multiplex assay results in amplifying all three target DNA sequences within the same tube, the costs, labor, and time required for sample preparation are reduced. This assay therefore provides an opportunity for more rapid diagnosis of contagious *Mycoplasma* pathogens and the reduction of risk for false positive results caused by *A. laidlawii*. In particular, the assay can be used as a first pass identification method for making preliminary decisions on mycoplasma diagnosis, thereby minimizing the risk of pathogen spread within dairy herds.

## Supplemental Information

10.7717/peerj.11881/supp-1 Supplemental Information 1 Multiple sequence alignments of 16S rRNA (118 bp), *rpoB* (232 bp) and I6S-23S ITS (108 bp) DNA sequences targeting the *Mycoplasma* genus, *M. bovis* and *A. laidlawii*, respectively. Positions identical to the first sequence are indicated by dots and gaps indicated by dashes. The primers and probe (reverse compliment) binding sequences are underlined and highlighted. The *rpoB*, 16S rRNA genes, and ITS regions used for alignment are from the following strains: *M. bovis* strain PG45 (ATCC 25523, NC_014760), *M. californium* strain ST-6 (ATCC 33461, NZ_CP007521), *M. bovigenitalium* strain HAZ (ATCC 19852, AP017902), *M. canadense* strain HAZ 360_1 (ATCC 29410, NZ_AP014631), *M. alkalescens* 14918 (ATCC 29103, NZ_AMWK01000000) and *A. laidlawii* PG-8A (ATCC 23206, NC_010163)

10.7717/peerj.11881/supp-2 Supplemental Information 2 Standard curves for the 16S rRNA, *rpoB* and 16S-23S ITS TaqMan assays preformed individually (singleplex). The standard curves were constructed with 10-fold serial dilutions of *M. bovis* ATCC 25523 (16S rRNA and *rpoB* assays), *M. bovigenitalium* ATCC 19852 (16S rRNA assay), *M. californicum* ATCC 33461 (16S rRNA assay), and *A. laidlawii* ATCC 23206 (ITS assay) gDNA, ranging from between approximately 5 fg to 5 ng gDNA. Results shown are from a single run with each dilution tested in triplicate. The R^2^ value was 0.99 for the standard curve of each target (16S rRNA, *rpoB* and ITS). Error bars indicate standard deviation (±) based on the results for three replicates.

10.7717/peerj.11881/supp-3 Supplemental Information 3 qPCR standard curves. Raw data files of singplex and multiplex assays, limit of detection determination in milk, and mixed-species qPCR detection

## Additional Information and Declarations

### Data Availability

The following information was supplied regarding data availability: The data are available in the Supplemental Files.
