# Supplementary material for: Development of a multiplex qPCR assay for the simultaneous detection of Mycoplasma bovis, Mycoplasma species, and Acholeplasma laidlawii in milk
Source: PeerJ. 2021 Aug 12;9:e11881. doi: 10.7717/peerj.11881 (PMC8364749; doi:10.7717/peerj.11881)
Supplement: Supplemental Information 1 — Positions identical to the first sequence are indicated by dots and gaps indicated by dashes. The primers and probe (reverse compliment) binding sequences are underlined and highlighted. The rpoB, 16S rRNA genes, and ITS regions used for alignment are from the following strains: M. bovis strain PG45 (ATCC 25523, NC_014760), M. californium strain ST-6 (ATCC 33461, NZ_CP007521), M. bovigenitalium strain HAZ (ATCC 19852, AP017902), M. canadense strain HAZ 360_1 (ATCC 29410, NZ_AP014631), M. alkalescens 14918 (ATCC 29103, NZ_AMWK01000000) and A. laidlawii PG-8A (ATCC 23206, NC_010163) [file peerj-09-11881-s001.pdf]

16S rRNA DNA sequences

```
M. bovis           CGAGCGCAACCCTTATCCTTAGTTACTACC--ATTTAGTTGAGCACTCTAAGGAGACTGCCCGAG-TAATCGGGAGGAAGGTGGGGACGACGTCAAATCATCATGCCTCTTACGAGTGGGG 118
M. californicum    ............C.G.............A.--.............................T...-.......................................................
M. bovigenitalium  ............................A.--...C..............G..............-.......................................................
M. alkalescens     ............C....T..........A.GAG.CAT..C...G......GA..T......T.G.-...CT................T.................................
M. arginini        ............C....T..........A.GAG.CAT..C...G......GA..T......T.G.-...CT................T.................................
M. canadense       ............C....T..........A.GAG.CAT..C...G......GA..T......T.G.-...CT................T.................................
A. laidlawii       ................TGC.......C.T.--...A.....G.G......GC.........A.T.A...ATT...............T...................C....T..------
```

rpoB DNA sequences

```
M. bovis           TTTCAGCCGCTAACTTCAGAGCAAGACAAGAGGGAAAAGTTGAATATGTTGATGGCGCCAAAATTAAAATTAGGAATAATAAAGGCACATTAGATACATATAATTTAAAAAA 112
M. californicum    ----------------------------------------------------------------------------------------------------------------
M. bovigenitalium  ----------------------------------------------------------------------------------------------------------------
M.alkalescens      --------------------------------------------------------------------------------------------.....T...T......G..C
M.arginini         ----------------------------------------------------------------------------------------------------------------
M.canadense        -------------------------------------------------------------------------------------------....T.....T......G..C
A. laidlawii       ----------------------------------------------------------------------------------------------------------------

M.bovis            CTTCCAACGTTCAAATCAAGATACAGTTATTCACCAAAAACCTATTATTAAAGTT-GGTCAAGAAGTTGCTAAAGGCGATTTATTAGTTGATGGGTCAAGCTTCAAGGATGGGGAACTTGC 232
M.californicum     -..............C...........AG.......G......T.AG......AA-.......TT...AAA.....T......C.T........C...TCA..T..AA....T...-----
M.bovigenitalium   ...............C............G.............AC..G....G.AA-......C.....AAA.....T......C.T........CG..TCA..T........A..------
M.alkalescens      A..TG...............GA..CT.A.....T...GTT..GC..G.....T.A-............AGAGTT..T.................---------------------------
M.arginini         ----------..C..C....G....T.A.........GT...-....G....AC.AAA..........AAAGCT..T.................---------------------------
M.canadense        A..TG..A.A.....C....GG...T.A..C..T...GTG..GC..G.....T.A-AA.......A..AAAGCG..T.................---------------------------
A. laidlawii       ------------------------------------------------------.-...AC...............---------------------------------------------
```

16S-23S ITS DNA sequences

```
A. laidlawii       AAGTGGGCAATACCCAACGCCGGTGGCCTAACCCGAAAGGGAGGGAGCCGTCTAAGGTAGGGTCCATGATTGGGGTTAAGTCGTAACAAGGTATCCCTACGGGAACGT 108
M. bovis           ------------------------------------------------------------------...C...............................A......
M. californicum    ------------------------------------------------------------------...C...............................A......
M. bovigenitalium  ------------------------------------------------------------------...C...............................A......
M. alkalescens     ---------------------------------------.....CGA...C..........ACTGG...C......G........................A......
M. arginini        -----..T.........A.T....TAG......T----C.....CGA...C..........ACTGG...C......G........................A......
M. canadense       -----..T.........A.T....TAG......T----C.....CGA...C..........ACTGG...C......G........................A......
```
