# Supplementary figures and images for: Development of a multiplex qPCR assay for the simultaneous detection of Mycoplasma bovis, Mycoplasma species, and Acholeplasma laidlawii in milk
Source: PeerJ. 2021 Aug 12;9:e11881. doi: 10.7717/peerj.11881 (PMC8364749; doi:10.7717/peerj.11881)

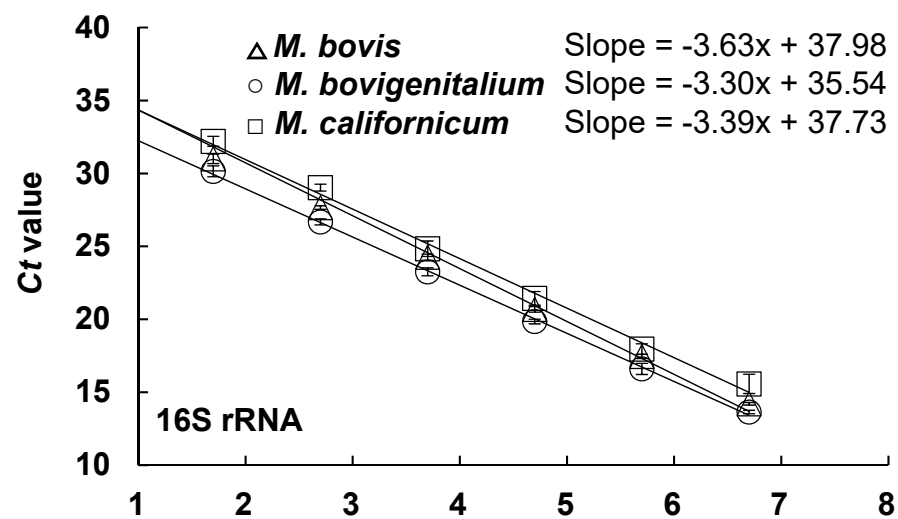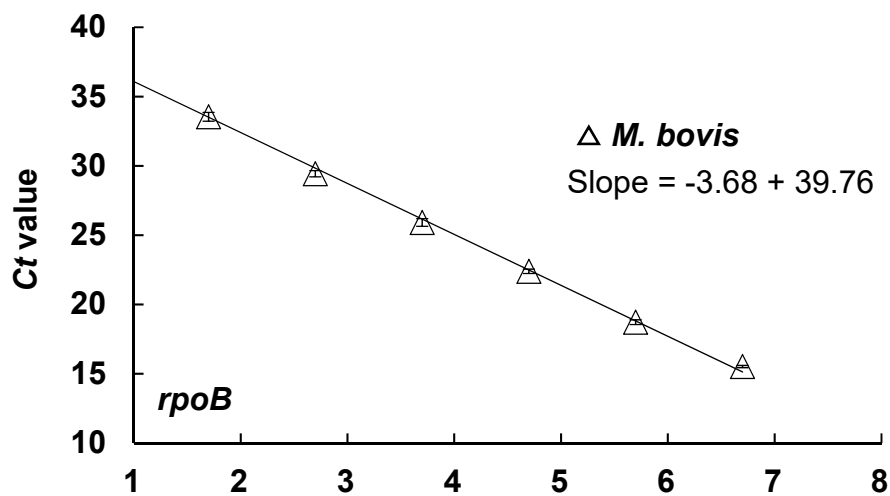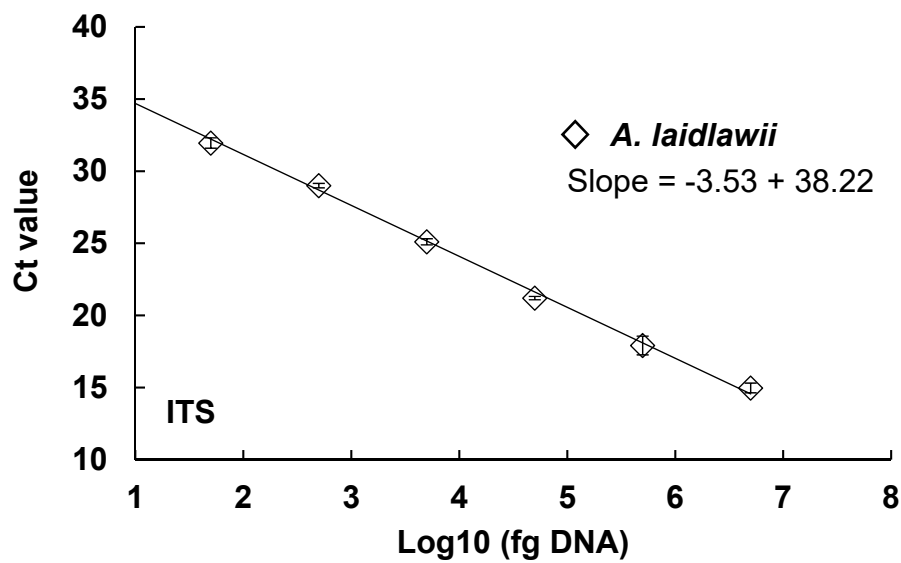

Supplement: Supplemental Information 2 — The standard curves were constructed with 10-fold serial dilutions of M. bovis ATCC 25523 (16S rRNA and rpoB assays), M. bovigenitalium ATCC 19852 (16S rRNA assay), M. californicum ATCC 33461 (16S rRNA assay), and A. laidlawii ATCC 23206 (ITS assay) gDNA, ranging from between approximately 5 fg to 5 ng gDNA. Results shown are from a single run with each dilution tested in triplicate. The R2 value was 0.99 for the standard curve of each target (16S rRNA, rpoB and ITS). Error bars indicate standard deviation (±) based on the results for three replicates. [file peerj-09-11881-s002.pdf]
